# Microbial ecological memory of recurrent drought enhances host stress tolerance through enrichment of plant‐beneficial bacteria

**DOI:** 10.1002/imo2.70110

**Published:** 2026-06-15

**Authors:** Chengguang Xing, Dandan Zhou, Dongwei Hou, Chunyi Lei, Zhiwei Liu, Jinhui Liu, Chaojian Hu, Jinye Liu, Shuzhen Huang, Yong Huo, Mingrui Zhou, Weiqiu Liu

**Affiliations:** ^1^ Guangdong Provincial Key Laboratory of Plant Stress Biology, School of Ecology Sun Yat‐sen University Shenzhen China; ^2^ State Key Laboratory of Biocontrol Sun Yat‐sen University Guangzhou China; ^3^ Guangdong Province Key Laboratory for Aquatic Economic Animals, School of Marine Sciences Sun Yat‐sen University Zhuhai China; ^4^ Department of Scientific Research and Education Heishiding Nature Reserve Zhaoqing China

## Abstract

Recurrent desiccation induced niche‐specific microbial ecological memory in the green part of *Calohypnum plumiforme*, marked by enrichment of plant‐beneficial bacteria, especially *Methylobacterium* and *Sphingomonas*, and functional shifts toward symbiosis and stress protection, potentially enhancing host tolerance within a “holobiont ecology memory” framework.

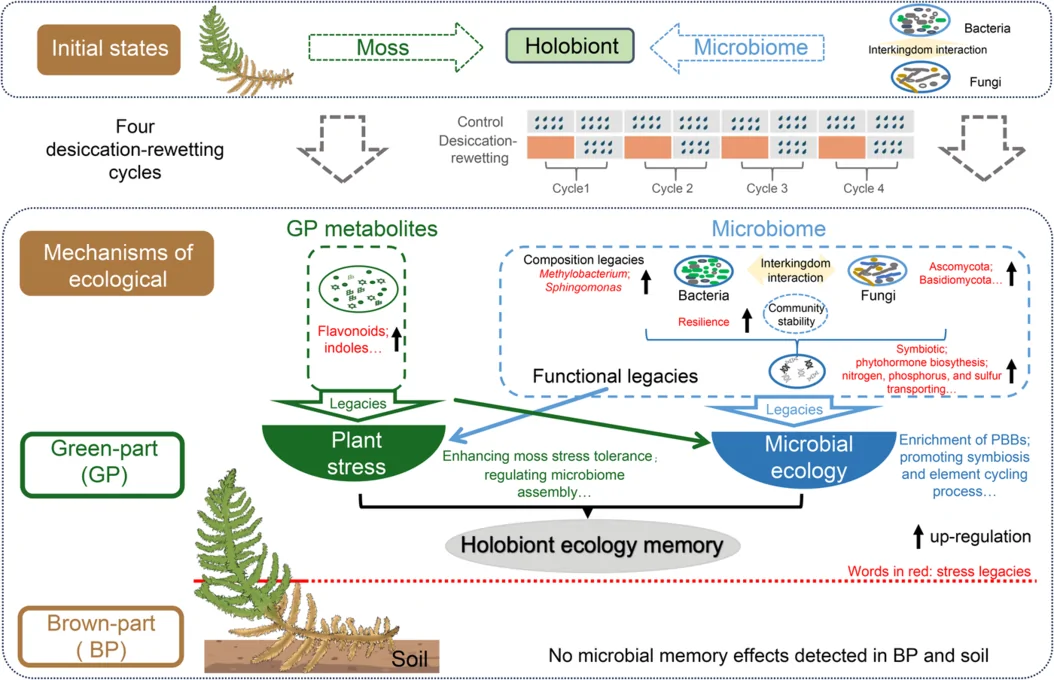

## ETHICS STATEMENT

No humans or animals were involved in this study.

To the Editor,

Ecological memory, the capacity of past disturbances to shape subsequent ecological responses, is increasingly recognized as central to understanding ecosystem stability and functionality under increasingly frequent climate extremes [1, 2, 3, 4]. In microbial ecology, recurrent drought is known to leave persistent legacies in soil communities and ecosystem functioning [2, 3, 5]. Yet whether comparable memory effects occur in plant‐associated microbiomes, and whether they become integrated with host stress memory at the holobiont level, remains largely unresolved. This question is especially important in the phyllosphere, where microbial communities experience repeated fluctuations in hydration, nutrients, and host‐derived metabolites, and where stress responses may emerge not only from microbial acclimation alone, but also from feedback between the host and its microbiome [6, 7, 8]. Within this perspective, we use the term “holobiont ecological memory” to describe a coupled stress‐memory framework in which host metabolic reprogramming and microbial community reassembly mutually reinforce each other under recurrent stress. Bryophytes provide an especially informative system for addressing this question because their exposed aerial tissues repeatedly undergo dehydration and rehydration, and their stress performance is tightly linked to associated microbial partners [7, 8, 9, 10, 11].

Here, using the desiccation‐tolerant moss *Calohypnum plumiforme*, we tested whether recurrent desiccation‐rewetting (DRW) generates a persistent microbial legacy in the phyllosphere and whether enrichment of plant‐beneficial bacteria (PBBs) constitutes a central biotic component of that legacy. We considered PBBs to be strong candidate legacies because plants under stress can selectively recruit beneficial microbes through altered metabolite chemistry, a process often described as a microbial “cry for help,” and repeated stress may strengthen this selective filtering and retention over time [7, 12, 13].

Our results showed that repeated DRW cycles generated a strong and niche‐specific microbial ecological memory in the green part (photosynthetically active phyllosphere), whereas the brown part and surrounding soil exhibited much weaker responses. This memory was expressed as progressive community stabilization, selective enrichment of PBBs, and coordinated functional and metabolic shifts consistent with enhanced stress responsiveness. Together, these findings suggest that recurrent drought can leave a biologically meaningful microbiome legacy in plant aerial tissues and that this legacy is best understood as part of a broader “holobiont ecological memory” framework.

## 1 NICHE‐SPECIFIC ECOLOGICAL MEMORY EMERGED IN THE PHYLLOSPHERE COMPARTMENT

We subjected *C. plumiforme* to four sequential DRW cycles and profiled bacterial and fungal communities in the green part (GP), brown part (BP), and soil, tracking physiological dynamics every 2 days (Figure S1). *C. plumiforme* exhibited typical desiccation stress followed by rapid recovery upon rewetting (Figure S2). Across compartments, the strongest treatment effects were consistently concentrated in GP. Repeated DRW significantly altered both bacterial and fungal community composition in GP, whereas BP and soil showed comparatively weak restructuring (Figure 1A, Figures S3–S6, and Table S1). This pattern indicates that microbial ecological memory did not emerge as a system‐wide response, but rather as a niche‐specific legacy in the living and directly exposed green tissue. This compartment specificity may reflect niche‐dependent adaptation, as GP is directly exposed to atmospheric drying and may experience stronger host‐mediated selection, whereas BP and soil are likely more buffered against rapid dehydration‐rehydration stress [7, 8, 9, 10].

**Figure 1:**
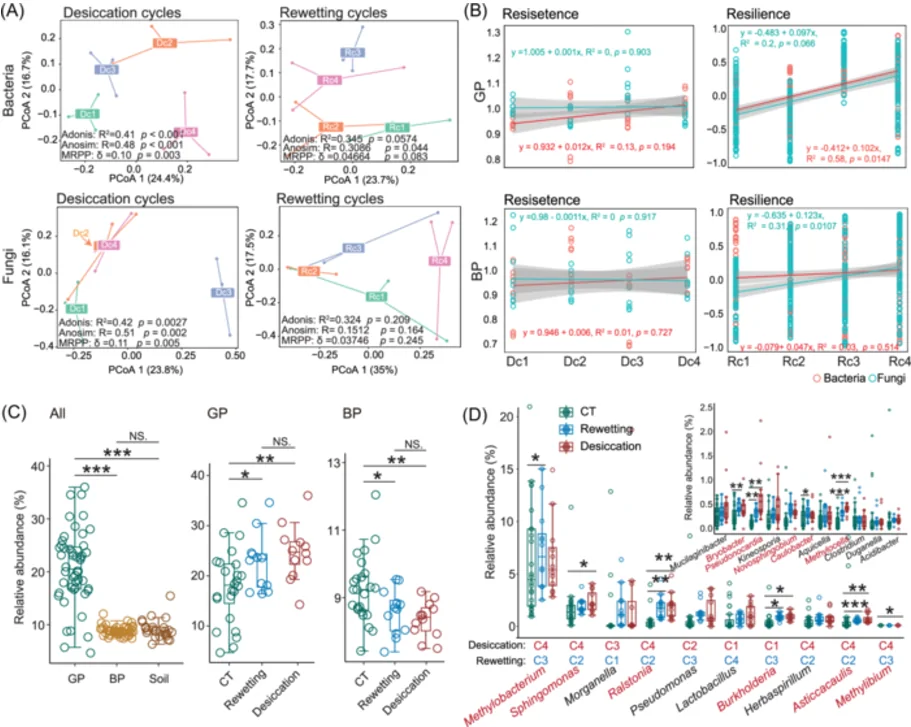
Recurrent drought generates a niche‐specific microbial ecological memory and enriches plant‐beneficial bacteria in GP of *Calohypnum plumiforme*. (A) Based on Bray–Curtis dissimilarity, PCoA for bacterial and fungal communities in GP under four desiccation‐rewetting cycles, respectively. Three non‐parametric multivariate analyses were conducted among different cycles: Adonis, ANOSIM, and MRPP. (B) Analysis of community stability (resistance and resilience) in response to four desiccation‐rewetting cycles in GP and BP. (C) Relative abundance of PBB across treatments in GP and BP. (D) The 20 most abundant PBB genera under CT and desiccation–rewetting cycles (genera ranked 11–20 are shown in the inset). Red‐colored genera indicate significant differences among treatments. C1–4 correspond to clusters 1–4 generated using Ward's clustering algorithm. Significant differences were determined using the nonparametric Kruskal–Wallis test. Asterisks indicate significance levels: *** represents *p* < 0.001, ** represents *p* < 0.01, * represents *p* < 0.05, and NS. represents *p* > 0.05. Adonis, permutational multivariate analysis of variance; ANOSIM, analysis of similarity; BP, brown parts; CT, control treatment; Dc1–4, desiccation cycle 1–4; Rc1–4, rewetting cycle 1–4; GP, green parts; MRPP, multiple response permutation procedure; PCoA, principal coordinates analysis; PBB, plant‐beneficial bacteria.

The GP microbiome also became progressively more stable under repeated stress. Across cycles, GP bacterial communities showed reduced compositional turnover and greater resilience following recurrent desiccation, while null‐model analysis indicated an increasing contribution of deterministic assembly, especially heterogeneous selection (Figure 1B, Figures S3 and S7, and Table S2). These results suggest that repeated drought history progressively filtered the GP microbiome toward a more stress‐adapted state. Given that GP is both metabolically active and directly exposed to rapid dehydration‐rehydration transitions, it may experience a stronger combination of abiotic filtering and host‐associated selection than BP and soil [7, 8, 9, 10].

This progressive stabilization was also accompanied by reorganization of bacterial–fungal interaction networks in GP. Under recurrent desiccation and rewetting, interkingdom networks remained dominated by positive associations, while the relative contribution of fungal versus bacterial nodes shifted across phases, with fungal nodes becoming more prominent during desiccation and bacterial nodes increasing during rewetting (Figures S8 and S9). Compared with the control treatment (CT) network background, these phase‐dependent changes suggest that recurrent drought history reshaped not only community assembly but also the balance of cross‐kingdom interactions, further supporting the view that ecological memory in GP involves coordinated restructuring at both the compositional and interaction‐network levels.

## 2 PLANT‐BENEFICIAL BACTERIA FORMED THE DOMINANT BIOTIC LEGACY

The clearest taxonomic signature of this microbial memory was the progressive enrichment of PBBs in GP. Among all sampled compartments, GP harbored the highest relative abundance of PBBs, and repeated DRW further increased their abundance specifically in this compartment, whereas PBB abundance declined in BP and changed little in soil (Figure 1C and Figures S10–S14). This contrasting pattern supports selective host‐associated retention rather than a generalized increase of beneficial taxa throughout the system.

Several well‐recognized beneficial genera contributed strongly to this pattern. In particular, *Methylobacterium* and *Sphingomonas* were among the dominant enriched taxa in GP, and multiple abundant PBB genera increased across repeated cycles (Figure 1D and Figures S12–S14). Notably, *Methylobacterium* displayed a relatively tolerant temporal pattern across both desiccation and rewetting phases, suggesting that it may represent a stable microbial legacy of recurrent drought. PBB abundance was also tightly associated with the overall bacterial community trajectory, indicating that these taxa were not minor responders but major contributors to microbiome reassembly.

This result provides a plausible bridge between microbiome memory and host stress performance. Many PBBs are known to support plant function through nutrient mobilization, stress protection, and phytohormone‐related effects [7, 12, 14, 15]. In addition, stress‐induced host filtering may preferentially retain these taxa across repeated cycles, making them plausible biotic legacies through which recurrent drought becomes biologically embedded in the phyllosphere microbiome [7]. In bryophytes, which lack vascular buffering and rely strongly on external water availability and microbially mediated nutrient processes, such beneficial partners may be especially important [9, 10]. We therefore interpret PBB enrichment as the dominant biotic legacy through which recurrent drought may strengthen phyllosphere stress responsiveness. Fungal responses were also detectable, but they were more variable and are best treated as supporting evidence rather than the central signal of ecological memory (Figures S6 and S15).

## 3 FUNCTIONAL AND HOST‐ASSOCIATED SHIFTS SUPPORTED A “HOLOBIONT ECOLOGICAL MEMORY” FRAMEWORK

Taxonomic shifts in GP were accompanied by pronounced functional reorganization. Shotgun metagenomic profiles from the fourth desiccation cycle (Dc4) diverged clearly from both the continuously watered control and the first desiccation cycle (Dc1), indicating that repeated stress generated a substantially stronger functional legacy than a single exposure (Figure 2A, Figures S16 and S17). Relative to Dc1, Dc4 exhibited broader enrichment of genes related to symbiotic interaction, signaling and cellular processing, secretion systems, trehalose‐associated processes, glycoside metabolism, and major element‐cycling pathways (Figure 2B–D and Figure S17). These functions are consistent with microbial persistence and interaction capacity under repeated dehydration.

**Figure 2:**
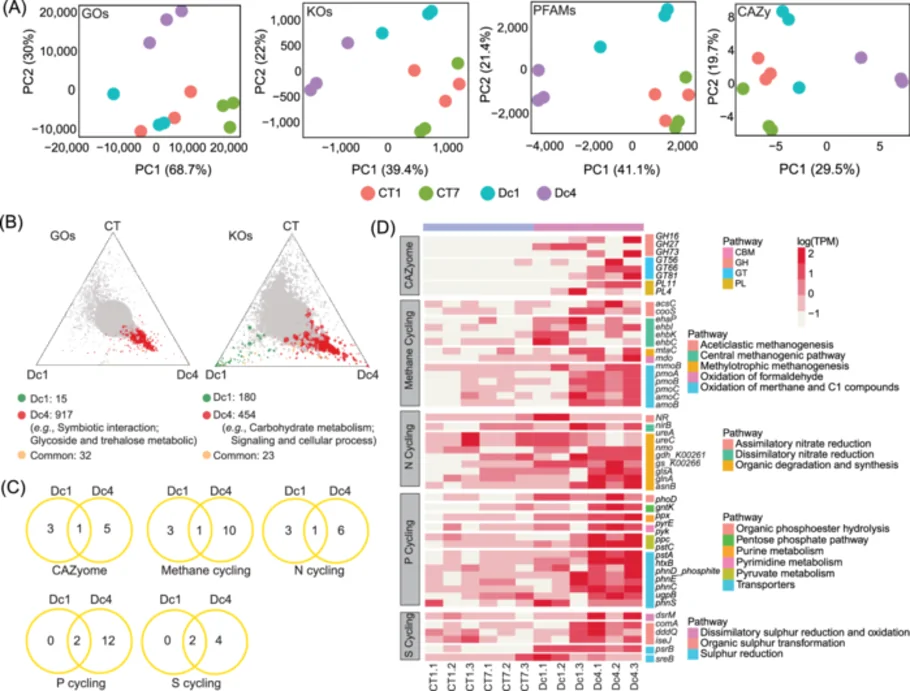
Functional and host‐associated shifts support a holobiont‐scale stress‐memory framework under recurrent drought in GP. (A) PCA of GOs, KOs, PFAMs, and CAZy profiles. (B) Ternary plots displaying differentially abundant functional genes (*p* < 0.05) between CT and desiccation cycle 1 (Dc1) and cycle 4 (Dc4). Statistical significance was determined using EdgeR's GLM approach. Each circle represents one functional gene, and the size of each circle represents its relative abundance. Gray circles represent non‐significantly different genes, whereas colored circle represents significantly different genes. (C) Venn diagrams showing shared and unique significantly different functional genes related to element cycling between Dc1 and Dc4 compared with CT. (D) Heatmap representing the abundance of genes involved in element cycling across CT, Dc1, and Dc4. CAZy, Carbohydrate‐Active Enzymes database; CBM, Carbohydrate‐Binding Module; CT, control treatment; Dc1, desiccation cycle 1; Dc4, desiccation cycle 4; GH, Glycoside Hydrolase; GT, Glycosyltransferase; GOs, Gene Ontology; KOs, KEGG Orthology; PCA, principal component analysis; PFAMs, Protein Families database; TPM, transcripts per million; PL, polysaccharide lyase.

Host metabolism also exhibited a parallel memory‐like signature. Moss metabolite profiles diverged after repeated DRW, with flavonoids, indoles, and carboxylic acids among the metabolites most strongly enriched under repeated stress (Figures S18 and S19). Exploratory structural equation modeling revealed putative links among PBB abundance, microbial functional shifts, and host stress‐memory metabolites (Figure S20). While these associations remain correlative, they align with a reciprocal regulatory framework wherein repeated desiccation orchestrates the coordinated adjustment of both host metabolic states and the phyllosphere microbiome.

Taken together, our data support the view that the microbial ecological memory observed here is best interpreted as part of a broader holobiont‐scale stress‐memory framework [6]. Importantly, however, our results do not imply that this phenomenon is restricted to mosses alone. Rather, the moss system provides a tractable and ecologically relevant model showing that recurrent drought can generate microbiome legacies in exposed plant‐associated aerial niches. This expands ecological memory theory beyond soil systems and suggests that phyllosphere microbiomes may represent an underappreciated component of plant resilience under repeated climate stress.

## 4 SHORT SUMMARY

In summary, recurrent desiccation generated a strong and niche‐specific microbial ecological memory in the phyllosphere of *C. plumiforme*. This memory was characterized by progressive community stabilization, selective enrichment of plant‐beneficial bacteria, and functional as well as metabolic legacies consistent with enhanced stress responsiveness. These results support a “holobiont ecological memory” framework in which recurrent drought couples microbial legacies with host stress‐memory traits under repeated stress. Our study thus extends ecological memory from soil microbial systems to plant‐associated aerial microbiomes and highlights phyllosphere microbiome legacies as a potential component of plant resilience under recurrent drought. Because these inferences are based mainly on community, metagenomic, metabolomic, and structural equation model associations, future inoculation experiments using defined bacterial consortia, especially involving *Methylobacterium* and *Sphingomonas*, will be necessary to establish direct causal contributions to host desiccation tolerance.

## AUTHOR CONTRIBUTIONS

**Chengguang Xing**: Writing—review and editing; writing—original draft; conceptualization; methodology; investigation; formal analysis; visualization. **Dandan Zhou**: Methodology; writing—review and editing. **Dongwei Hou**: Methodology; writing—review and editing. **Chunyi Lei**: Conceptualization. **Zhiwei Liu**: Methodology. **Jinhui Liu**: Investigation. **Chaojian Hu**: Methodology. **Jinye Liu**: Investigation. **Shuzhen Huang**: Investigation. **Yong Huo**: Investigation. **Mingrui Zhou**: Investigation. **Weiqiu Liu**: Writing—review and editing; conceptualization; supervision; project administration; funding acquisition.

## CONFLICT OF INTEREST STATEMENT

The authors declare no conflicts of interest.

## Supporting information


**Figure S1:** Experimental design and sampling scheme.
**Figure S2:** Physiological and environmental validation of control and repeated desiccation‐rewetting treatment.
**Figure S3:** Alpha diversity of bacterial and fungal communities across compartments and treatments.
**Figure S4:** Temporal species turnover in the brown‐part and soil microbiomes.
**Figure S5:** Principal coordinate analysis (PCoA) of bacterial and fungal communities across compartments and treatments, with expanded BP and soil view.
**Figure S6:** Beta diversity patterns and compositional stabilization of microbiomes across compartments.
**Figure S7:** Community assembly processes in green‐part, brown‐part, and soil microbiomes.
**Figure S8:** Temporal dynamics of bacterial‐fungal interkingdom network in GP under desiccation and rewetting treatment.
**Figure S9:** Temporal dynamics of bacterial‐fungal interkingdom network in GP under CT treatment.
**Figure S10:** Bacterial taxonomic composition at the phylum and genus levels across compartments and treatments.
**Figure S11:** Longitudinal clustering of bacterial genera during desiccation and rewetting phases.
**Figure S12:** Heatmaps and longitudinal shifts of desiccation‐ and rewetting‐responsive bacterial modules in GP.
**Figure S13:** Progressive increase of plant‐beneficial bacteria across desiccation cycles.
**Figure S14:** Correlation between plant‐beneficial bacteria and community dynamics.
**Figure S15:** Composition of fungal communities and desiccation‐responsive fungal taxa.
**Figure S16:** Functional alpha diversity of GP microbiomes across treatments.
**Figure S17:** Gene Ontology (GO) and KEGG pathway enrichment analysis for Dc4.
**Figure S18:** Heatmap of memory metabolites in GP.
**Figure S19:** PCA of metabolites.
**Figure S20:** Structural equation models (SEM) linking microbial and metabolic legacies under recurrent drought.

**Table S1:** PERMDISP tests showing within‐group dispersion differences among desiccation and rewetting cycles in bacterial and fungal communities of GP.
**Table S2:** Crossed linear mixed‐effects model results for bacterial and fungal resistance and resilience in GP and BP compartments along desiccation–rewetting cycles.

## Data Availability

All the sequencing data have been deposited in NCBI under submission numbers SRR38557756–SRR38558061 (https://www.ncbi.nlm.nih.gov/sra?linkname=bioproject_sra_all&from_uid=1465055), BioProject accession number PRJNA1465055 (https://www.ncbi.nlm.nih.gov/bioproject/PRJNA1465055). The data and scripts used are saved in GitHub https://github.com/XingChengguang/Microbial-ecological-memory-in-moss.git. Supplementary materials (methods, figures, graphical abstract, slides, videos, Chinese translated version, and updated materials) may be found in the online DOI or iMetaOmics Science http://www.imeta.science/imetaomics/.
