# Supplementary material for: Microbial ecological memory of recurrent drought enhances host stress tolerance through enrichment of plant‐beneficial bacteria
Source: IMetaOmics. 2026 Jun 15;3(3):e70110. doi: 10.1002/imo2.70110 (PMC13674387; doi:10.1002/imo2.70110)
Supplement: Supplementary file 1 [file IMO2-3-e70110-s002.docx]

Supporting information to

**Microbial ecological memory of recurrent drought enhances host stress tolerance through enrichment of plant-beneficial bacteria**

**Running title**: Phyllosphere microbial ecological memory under recurrent drought

Chengguang Xing^1, 2#^, Dandan Zhou^2, 3#^, Dongwei Hou^2, 3^, Chunyi Lei^4^, Zhiwei Liu^1, 2^, Jinhui Liu^1, 2^, Chaojian Hu^1, 2^, Jinye Liu^1, 2^, Shuzhen Huang^1, 2^, Yong Huo^1, 2^, Mingrui Zhou^1, 2^, Weiqiu Liu^1, 2*^

^1^Guangdong Provincial Key Laboratory of Plant Stress Biology, School of Ecology, Sun Yat-sen University, Shenzhen, 518107, China

^2^State Key Laboratory of Biocontrol, Sun Yat-sen University, Guangzhou, 510275, China

^3^Guangdong Province Key Laboratory for Aquatic Economic Animals, School of Marine Sciences, Sun Yat-sen University, Zhuhai, 519082, China

^4^Department of Scientific Research and Education, Heishiding Nature Reserve, Zhaoqing, 526500, China

^#^These authors contributed equally: Chengguang Xing, Dandan Zhou

^*^Correspondence: [lsslwq@mail.sysu.edu.cn (Weiqiu Liu)](mailto:xxx@edu.cn)

**METHODS**

**Experimental design, sampling, and measurement of moss and soil properties**

In February 2022, *Calohypnum plumiforme* were sampled from Heishiding Nature Reserve (23°25′−23°29′N, 111°49′−111°55′E; 400−500 meters above sea level. They were immediately transplanted to the bottom of a valley at the edge of the forest in Heishiding Natural Reserve and were planted in 48 trays (32 × 24 × 9 cm), with similar planting density to their natural communities. The gametophytes were acclimated until October 2022, with watering every two days with 300 mL for each tray to maintain hydration.

After acclimation, the 48 trays were moved to a screenhouse and randomly assigned to one of two experimental groups using a randomized block design for the main study conducted in autumn/winter 2022. The experimental design and group allocation were graphically illustrated and explained in Figure S1. Briefly, the first group served as the control treatment (CT), receiving 300 mL of water in each tray every two days throughout the entire experiment. The second group underwent four desiccation-rewetting cycles. Each cycle consisted of six days without water (desiccation) followed by six days of watering (rewetting). Previous experiments showed that six-day desiccation periods were sufficient to induce stress memory in moss with metabolic activity ceases entirely. During the desiccation cycles, no water was applied, and during the rewetting cycles, 300 mL of water was applied.

Chlorophyll fluorescence was measured using a FluorPen FP 110 fluorometer (Photon Systems Instruments, Drasov, Czech Republic). Sample leaves were dark-adapted for 15 min using detachable leaf clips to ensure complete relaxation of reaction centers. Measurements employed the QY protocol with manufacturer-calibrated pulse intensities: Flash pulse: 30% intensity (induction of minimal fluorescence, Fo), Super pulse: 80% intensity (saturation pulse for maximal fluorescence, Fm). Soil volumetric water content (VWC) was quantified using the AZS-2 Soil Moisture Probe (Aozuo Ecology Instruments Ltd., Beijing, China) paired with an SM-3 Handheld Reader. Tissue Water loss curves were fitted to a three-parameter exponential decay model:

**Equation1**

$$\text{Water content}\left( \text{\%} \right)\text{=a× e\textasciicircum(-DC × T) + b}$$

Where T (day) is the evaporative time, and a represents the initial water status amplitude, DC (decaying constant) quantifies the rate of water loss, with higher values indicating faster dehydration and lower drought tolerance, and b are coefficients.

Those three key measurements were monitored every two days. At the end of each desiccation cycle, significant leaf curling was observed (Figure S2A), with tissue water content ranging from 0.1% to 0.7% (Figure S2B), Fv/Fm values of 0.2−0.4 (Figure S2C), and soil volume water content ranging from 20% to 35% (Figure S2D). Water loss curves in moss, fitted using exponential decay regression, revealed a similar and slow drying process over the four rounds (Figure S2E). In each of the rewetting cycles, these indices quickly recovered to levels the same as those observed in the CT, indicating that the desiccation stress experienced by the moss had been alleviated (Figure S2B–E).

At each end of the phase (eight times), we sampled the green part (GP, living part, the top two to five centimeters of the moss), the brown part (BP, senescent part), and the surrounding soil. To avoid confounding effects of circadian rhythms, sampling was conducted at 14:00. The collected moss samples were washed quickly with distilled water, immediately placed in liquid nitrogen, and stored at −80 ^◦^C until further analysis. Similarly, surrounding soil samples were immediately frozen in liquid nitrogen and stored at −80 ^◦^C until further analysis.

**DNA extraction and amplicon sequencing and bioinformatic analysis**

The GP and BP of moss were ground using sterile mortars and pestles with liquid nitrogen. DNA was extracted using the PowerSoil DNA Isolation Kit (MO BIO Laboratories, Carlsbad, CA, USA) following the manufacturer’s instructions. DNA concentrations were measured on a Varian Eclipse fluorescence plate reader (Agilent, USA). Bacterial profiles are derived from PCR primers 515F (5'-GTG CCA GCM GCC GCG GTA A-3') and 806R (5'-GGA CTA CHV GGGTWT CTA AT-3') that span the hypervariable regions V3−V4 region of 16S rDNA gene and fungal profiles are based on ITS1 region amplified with primers ITS1-1F-F (5'-CTTGGTCATTTAGAGGAAGTAA-3') and ITS1-1F-R (5'-GCTGCGTTCTTCATCGATGC-3') [1]. Sequencing was carried out on the Illumina Novaseq6000 platform with a paired-end protocol in Novogene (Beijing, China). Sequence processing and quality control were conducted using the QIIME2 platform (v2023.2). Raw paired-end reads were denoised and filtered with the DADA2 plugin (v1.26.0). No trimming or truncation was applied to forward or reverse reads (--p-trim-left 0, --p-trunc-len 0), as preliminary quality assessments (FastQC reports) confirmed high sequencing quality across all cycles, and primer sequences had already been removed during demultiplexing. Denoising included error rate estimation, chimera removal via DADA2’s consensus method, and inference of exact amplicon sequence variants (ASVs). The resulting ASV table was filtered to remove singletons (ASVs with total counts < 5) Taxonomic classification was performed against with SILVA and UNITE reference databases [2–4]. Chloroplast and mitochondria sequences were removed.

Identification of plant-beneficial bacteria (PBBs) was based on the manually curated database [5]. Briefly, this database comprises bacterial genera with experimentally validated beneficial traits for plants, categorized as: biocontrol, plant growth promotion (*e.g.*, nutrient acquisition, phytohormone production), or enhanced plant stress tolerance, while excluding known pathogenic genera Taxonomic assignments were standardized using the SILVA reference databases [5]. Bacterial genera detected in the moss samples matching this database were classified as PBBs. Fungal functional guilds were inferred (guild assignments with confidence rankings “highly probable” and “probable” were retained) from the FUNGuild database [6]. Both bacterial and fungal alpha- and beta-diversity were calculated in QIIME [7]**.**

**Metagenome sequencing and data processing**

To further characterize the moss GP microbiome functional profile, we sequenced 12 DNA samples from desiccation cycle 1 (Dc1), desiccation cycle 4 (Dc4), and CT (CT1 and CT7, corresponding sampling points) by using the Illumina platforms with the paired-end protocol in Novogene (Beijing, China). Critically, DNA for metagenomic analysis originated from the same samples previously subjected to amplicon sequencing, ensuring direct comparability between taxonomic profiles and functional gene content. Raw sequences were quality-filtered using fastp, and sequences belonging to the moss genome were removed using bowtie2 [8,9]. The high-quality reads were assembled using Megahit, and open reading frames (ORFs) were predicted using Prodigal (v1.14.5) and clustered with a 0.95 similarity threshold using CD-HIT (v4.8.1) [10–12]. The function profiles, including KEGG Orthology (KO), Carbohydrate-Active Enzyme (CAZyome), Pfam, and Clusters of Orthologous Groups of proteins (COG) of GP microbiomes were determined using eggNOG databases (v6.0) by ggNOG-mapper v2.0.1 [13,14]. The alpha and beta index of functional diversity were calculated based on the rarefied table in QIIME [7]. The genes involved in methane, nitrogen, phosphorus, and sulfur cycling pathways were retrieved from manually curated methane cycling database, N cycling database, P cycling database, and S cycling database following their instruction scripts, respectively [15–18]. For cross-sample comparisons, functional gene abundances were normalized by subsampling all samples to the minimum sequencing depth using a rarefaction approach.

**Metabolites extraction identification and analysis**

According to the previous method, gametophyte surface microbes were thoroughly removed in 12 GP samples from Dc1, Dc4, and CT (CT1 and CT7) [19]. The cleaned samples were analyzed using liquid chromatography-mass spectrometry (LC-MS) with a Vanquish UHPLC system (Thermo Fisher, Germany) coupled with an Orbitrap Q Exactive HF-X mass spectrometer (Thermo Fisher, Germany) in Novogene (Beijing, China). Despite thorough surface cleaning, a small portion of metabolites may still originate from endophytic microorganisms. The raw data files were processed using the Compound Discoverer 3.3 (CD3.3, Thermo Fisher) and then were matched with the mzCloud, mzVault, and MassList databases to obtain accurate qualitative and relative quantitative results. These metabolites were annotated using the KEGG, HMDB, and LIPIDMaps databases [20–22]. Principal components analysis (PCA) and partial least squares discriminant analysis (PLS-DA) were performed at metaX [23]. The metabolites with VIP > 1 and *p*-value < 0.05 and fold change (FC) ≥ 2 or ≤ 0.5 were considered to be differential metabolites. Metabolites in high abundance significantly changed in Dc4 compared to Dc1 were identified as stress memory metabolites.

**Statistical analysis**

The effects of multiple factors on microbial alpha diversity were tested with a linear model (LMM) based on the richness index using the R package “lme4”, and significance was assessed using ANOVA [24]. At all sample levels, the relative contribution of different variables to community dissimilarity was tested with permutational multivariate analysis of variance (PERMANOVA) based on Bray-Curtis distances matrices using the Adonis function (R package “vegan”). Further, to evaluate whether the significant PERMANOVA results were influenced by differences in within-group multivariate dispersion, PERMDISP analysis was performed based on Bray–Curtis dissimilarities using the Betadisper function in the “vegan” package. Principal Coordinate Analysis (PCoA) was analyzed based on Bray-Curtis distances using the pcoa function from the “ape” package [25]. Beta diversity into species turnover and nestedness components was partitioned using “betapart” packages in R [26].

To assess the relative importance of determinism and stochasticity in microbial community assembly, the beta Nearest Taxon Index (βNTI) was calculated using a null model (999 randomizations). Further, deterministic and stochastic processes were partitioned into five ecological processes based on both βNTI and Bray-Curtis-based Raup-Crick Index (RCBray) values [27,28].

To evaluate the stability of the microbial community under different desiccation cycles, we adopted the approach proposed by Shade et al. and Gao et al., which involves assessing resistance and resilience by calculating pairwise community dissimilarity [29,30]. To characterize the ecological resistance to desiccation stress in each desiccation-rewetting treatment, we modified it by calculating pairwise community dissimilarity before desiccation and after each desiccation cycle, as outlined in Equation 1. For the resistance of Dc1, we calculated the dissimilarity between the microbial community at the initial time point and Dc1.

**Equation2**

$$\text{Resistence}\left( \text{Dcn} \right)\text{=}\text{Bray}\text{-}\text{Curtis}\text{ (}\text{Rcn}\text{-1, }\text{Dcn}\text{)}$$

Where Dcn represents the desiccation cycle n-th. Rcn-1 represents rewetting cycle (n-1)-th.

The ecological resilience to rewetting was evaluated by comparing the compositional dissimilarity between the control group and the microbial community after rewetting from desiccation stress, as described in Equation 2.

**Equation3**

$$\text{Resilience}\left( \text{Rcn} \right)\text{=}\text{Bray}\text{-}\text{Curtis}\text{ (}\text{Rcn}\text{, }\text{CTn}\text{)}$$

Where Rcn represents the rewetting cycles n. CTn represents the CT treatment group at the corresponding sampling time at rewetting cycles n.

To evaluate changes in resistance and resilience along the desiccation-rewetting cycles for each compartment, all 3 × 3 pairwise Bray-Curtis dissimilarities among the three biological replicates were retained for visualization, while statistical inference was performed using crossed linear mixed-effects models to account for the non-independence among pairwise dissimilarities. Specifically, resistance or resilience was modeled as a function of desiccation-rewetting cycles, with the identities of the two samples involved in each Bray-Curtis dissimilarity included as crossed random effects:

**Equation4**

$$\text{RS}\text{\textasciitilde Cycles+(1｜}\text{sample}\text{\_i)+}\text{ }\text{(1| sample\_j)}$$

where RS denotes the resistance or resilience value, cycles denotes the desiccation-rewetting cycles, and sample_i and sample_j denote the two sample identities used to calculate each Bray-Curtis dissimilarity. The fixed-effect equation, the *p* value for the fixed effect of cycles, and the marginal R² of the mixed model were reported in the main figure. Full mixed-model statistics were provided in the Table S2 [24].

Microbial interkingdom network analysis at bacterial and fungal genera level was performed using the “corr. Test” function in the package “psych” in R based on Spearman correlation. Both bacterial and fungal genera present in at least ten samples were retained for the network analysis. The *p* values were adjusted using the false discovery rate (FDR) method. A cutoff for the Spearman’s correlation coefficient (ρ) of higher than 0.8 or lower than –0.8 with *p* < 0.05 was used for network construction. The networks were visualized in Gephi.

The Kruskal-Wallis test was performed to evaluate the PBBs difference among different compartments and treatments. To further identify coherent patterns of microbial response to desiccation-rewetting cycles, we performed hierarchical clustering (Ward’s algorithm) on relative abundance changes with the hclust function in R. Differential abundance analysis of fungi and microbial function was performed using EdgeR’s generalized linear model (GLM) approach, with *p* < 0.05 using the Benjamini-Hochberg method adjustments. Heatmap and ternary plots were conducted using the “pheatmap” package and “ggtern” in R, respectively [31]. PCA analysis was performed to reveal the beta diversity of function genes.

To evaluate the influence of microbial composition and function on the formation of moss memory metabolites under desiccation-rewetting cycles, structural equation modeling (SEM) analyses were performed to examine the relationships among treatments, cycles, PBBs, desiccation-rewetting responsive fungi, and microbial functional genes (as captured by the PCA axis based on KOs and GOs), and memory metabolites using “plspm” packages in R. In the SEM results, the β values as standardized path coefficients, quantifying the magnitude and direction of direct effects between variables. Similarly, to further elucidated the moss influence on microbiome memory formation, another SEM was performed to examine the relationships among treatments, cycles, metabolites (as captured by the PCA axis), and microbial legacies in terms of PBBs, differentially abundant fungi and functional genes involved in element cycling.

**REFERENCES**

1. Caporaso, J. Gregory, Christian L. Lauber, William A. Walters, Donna Berg-Lyons, Catherine A. Lozupone, Peter J. Turnbaugh, Noah Fierer, Rob Knight. 2011. “Global patterns of 16S rRNA diversity at a depth of millions of sequences per sample.” *Proceedings of the National Academy of Sciences* 108: 4516–4522. <https://doi.org/10.1073/pnas.1000080107>

2. Callahan, Benjamin J., Paul J. McMurdie, Michael J. Rosen, Andrew W. Han, Amy Jo A. Johnson, Susan P. Holmes. 2016. “DADA2: high-resolution sample inference from Illumina amplicon data.” *Nature Methods* 13: 581–583. <https://doi.org/10.1038/nmeth.3869>

3. Quast, Christian, Elmar Pruesse, Pelin Yilmaz, Jan Gerken, Timmy Schweer, Pablo Yarza, Jörg Peplies, Frank Oliver Glöckner. 2013. “The SILVA ribosomal RNA gene database project: improved data processing and web-based tools.” *Nucleic Acids Research* 41: 590–596. <https://doi.org/10.1093/nar/gks1219>

4. Abarenkov, Kessy, R. Henrik Nilsson, Karl-Henrik Larsson, Andy F S. Taylor, Tom W May, Tobias Guldberg Frøslev, Julia Pawlowska, et al. 2024. “The UNITE database for molecular identification and taxonomic communication of fungi and other eukaryotes: sequences, taxa and classifications reconsidered.” *Nucleic Acids Research* 52: 791–797. <https://doi.org/10.1093/nar/gkad1039>

5. Li, Pengfa, Leho Tedersoo, Thomas W. Crowther, Alex J. Dumbrell, Francisco Dini-Andreote, Mohammad Bahram, Lu Kuang, et al. 2023. “Fossil-fuel-dependent scenarios could lead to a significant decline of global plant-beneficial bacteria abundance in soils by 2100.” *Nature Food* 4: 996–1006. <https://doi.org/10.1038/s43016-023-00869-9>

6. Nguyen, Nhu H., Zewei Song, Scott T. Bates, Sara Branco, Leho Tedersoo, Jon Menke, Jonathan S. Schilling, Peter G. Kennedy. 2016. “FUNGuild: An open annotation tool for parsing fungal community datasets by ecological guild.” *Fungal Ecology* 20: 241–248. <https://doi.org/10.1016/j.funeco.2015.06.006>

7. Bolyen, Evan, Jai Ram Rideout, Matthew R. Dillon, Nicholas A. Bokulich, Christian C. Abnet, Gabriel A. Al-Ghalith, Harriet Alexander, et al. 2019. “Reproducible, interactive, scalable and extensible microbiome data science using QIIME 2.” *Nature Biotechnology* 37: 852–857. <https://doi.org/10.1038/s41587-019-0209-9>

8. Chen, Shifu. 2023. “Ultrafast one-pass FASTQ data preprocessing, quality control, and deduplication using fastp.” *iMeta* 2: e107. <https://doi.org/10.1002/imt2.107>

9. Langmead, Ben, Steven L. Salzberg. 2012. “Fast gapped-read alignment with Bowtie 2.” *Nature Methods* 9: 357–359. <https://doi.org/10.1038/nmeth.1923>

10. Li, Dinghua, Ruibang Luo, Chi-Man Liu, Chi-Ming Leung, Hing-Fung Ting, Kunihiko Sadakane, Hiroshi Yamashita, Tak-Wah Lam. 2016. “MEGAHIT v1.0: A fast and scalable metagenome assembler driven by advanced methodologies and community practices.” *Methods* 102: 3–11. <https://doi.org/10.1016/j.ymeth.2016.02.020>

11. Hyatt, Doug, Gwo-Liang Chen, Philip F. LoCascio, Miriam L. Land, Frank W. Larimer, Loren J. Hauser. 2010. “Prodigal: prokaryotic gene recognition and translation initiation site identification.” *BMC Bioinformatics* 11: 119. <https://doi.org/10.1186/1471-2105-11-119>

12. Fu, Limin, Beifang Niu, Zhengwei Zhu, Sitao Wu, Weizhong Li. 2012. “CD-HIT: accelerated for clustering the next-generation sequencing data.” *Bioinformatics* 28: 3150–3152. <https://doi.org/10.1093/bioinformatics/bts565>

13. Huerta-Cepas, Jaime, Damian Szklarczyk, Davide Heller, Ana Hernández-Plaza, Sofia K. Forslund, Helen Cook, Daniel R. Mende, et al. 2019. “eggNOG 5.0: a hierarchical, functionally and phylogenetically annotated orthology resource based on 5090 organisms and 2502 viruses.” *Nucleic Acids Research* 47: 309–314. <https://doi.org/10.1093/nar/gky1085>

14. Cantalapiedra, Carlos P., Ana Hernández-Plaza, Ivica Letunic, Peer Bork, Jaime Huerta-Cepas. 2021. “eggNOG-mapper v2: functional annotation, orthology assignments, and domain prediction at the metagenomic scale.” *Molecular Biology and Evolution* 38: 5825–5829. <https://doi.org/10.1093/molbev/msab293>

15. Qian, Lu, Xiaoli Yu, Jiayin Zhou, Hang Gu, Jijuan Ding, Yisheng Peng, Qiang He, et al. 2022. “MCycDB: a curated database for comprehensively profiling methane cycling processes of environmental microbiomes.” *Molecular Ecology Resources* 22: 1803–1823. <https://doi.org/10.1111/1755-0998.13589>

16. Tu, Qichao, Lu Lin, Lei Cheng, Ye Deng, Zhili He. 2019. “NCycDB: a curated integrative database for fast and accurate metagenomic profiling of nitrogen cycling genes.” *Bioinformatics* 35: 1040–1048. <https://doi.org/10.1093/bioinformatics/bty741>

17. Yu, Xiaoli, Jiayin Zhou, Wen Song, Mengzhao Xu, Qiang He, Yisheng Peng, Yun Tian, et al. 2021. “SCycDB: A curated functional gene database for metagenomic profiling of sulphur cycling pathways.” *Molecular Ecology Resources* 21: 924–940. <https://doi.org/10.1111/1755-0998.13306>

18. Zeng, Jiaxiong, Qichao Tu, Xiaoli Yu, Lu Qian, Cheng Wang, Longfei Shu, Fei Liu, et al. 2022. “PCycDB: a comprehensive and accurate database for fast analysis of phosphorus cycling genes.” *Microbiome* 10: 101. <https://doi.org/10.1186/s40168-022-01292-1>

19. Xiong, Chao, Brajesh K. Singh, Ji-Zheng He, Yan-Lai Han, Pei-Pei Li, Li-Hua Wan, Guo-Zhong Meng, et al. 2021. “Plant developmental stage drives the differentiation in ecological role of the maize microbiome.” *Microbiome* 9: 171. <https://doi.org/10.1186/s40168-021-01118-6>

20. Kanehisa, Minoru, Miho Furumichi, Yoko Sato, Masayuki Kawashima, Mari Ishiguro-Watanabe. 2023. “KEGG for taxonomy-based analysis of pathways and genomes.” *Nucleic Acids Research* 51: 587–592. <https://doi.org/10.1093/nar/gkac963>

21. Wishart, David S., AnChi Guo, Eponine Oler, Fei Wang, Afia Anjum, Harrison Peters, Raynard Dizon, et al. 2022. “HMDB 5.0: the human metabolome database for 2022.” *Nucleic Acids Research* 50: 622–631. <https://doi.org/10.1093/nar/gkab1062>

22. Conroy, Matthew J., Robert M. Andrews, Simon Andrews, Lauren Cockayne, Edward A Dennis, Eoin Fahy, Caroline Gaud, et al. 2024. “LIPID MAPS: update to databases and tools for the lipidomics community.” *Nucleic Acids Research* 52: 1677–1682. <https://doi.org/10.1093/nar/gkad896>

23. Wu, Qing, Zhibin Ning, Ailing Zhang, Xu Zhang, Zhongzhi Sun, Daniel Figeys. 2025. “Operational taxon-function framework in metaX: unveiling taxonomic and functional associations in metaproteomics.” *Analytical Chemistry* 97: 9739–9747. <https://doi.org/10.1021/acs.analchem.4c06645>

24. Bates, Douglas, Martin Mächler, Ben Bolker, Steve Walker. 2015. “Fitting linear mixed-effects models using lme4.” *Journal of Statistical Software* 67: 1–48. <https://doi.org/10.18637/jss.v067.i01>

25. Paradis, Emmanuel, Julien Claude, Korbinian Strimmer. 2004. “APE: analyses of Phylogenetics and Evolution in R language.” *Bioinformatics* 20: 289–290. <https://doi.org/10.1093/bioinformatics/btg412>

26. Baselga, Andrés, C. David L. Orme. 2012. “betapart: an R package for the study of beta diversity.” *Methods in Ecology and Evolution* 3: 808–812. <https://doi.org/10.1111/j.2041-210X.2012.00224.x>

27. Jiao, Shuo, Yunfeng Yang, Yiqin Xu, Jie Zhang, Yahai Lu. 2020. “Balance between community assembly processes mediates species coexistence in agricultural soil microbiomes across eastern China.” *The ISME Journal* 14: 202–216. <https://doi.org/10.1038/s41396-019-0522-9>

28. Zhou, Jizhong, Ye Deng, Ping Zhang, Kai Xue, Yuting Liang, Joy D. Van Nostrand, Yunfeng Yang, et al. 2014. “Stochasticity, succession, and environmental perturbations in a fluidic ecosystem.” *Proceedings of the National Academy of Sciences* 111: E836–E845. <https://doi.org/10.1073/pnas.1324044111>

29. Shade, Ashley, Hannes Peter, Steven D. Allison, Didier Baho, Mercé Berga, Helmut Buergmann, David H. Huber, et al. 2012. “Fundamentals of microbial community resistance and resilience.” *Frontiers in Microbiology* 3: 417. <https://doi.org/10.3389/fmicb.2012.00417>

30. Gao, Cheng, Ling Xu, Liliam Montoya, Mary Madera, Joy Hollingsworth, Liang Chen, Elizabeth Purdom, et al. 2022. “Co-occurrence networks reveal more complexity than community composition in resistance and resilience of microbial communities.” *Nature Communications* 13: 3867. <https://doi.org/10.1038/s41467-022-31343-y>

31. Hamilton, Nicholas E., Michael Ferry. 2018. “ggtern: ternary diagrams using ggplot2.” *Journal of Statistical Software, Code Snippets* 87: 1–17. <https://doi.org/10.18637/jss.v087.c03>

**Figure S1 Experimental design and sampling scheme.** (A) Illustration of the *C. plumiforme* transplanting and acclimation process. Mosses were maintained in optimal conditions to mitigate the potential effects of prior stress history before exposure to the four desiccation-rewetting cycles. (B) Schematic representation of the control treatment (CT) and the desiccation-rewetting cycles design. Each cycle included approximately six days of desiccation stress followed by six days of rewetting, repeated over four cycles. During the desiccation phase, no water was provided, while during the rewetting phase, 300 ml of water was administered to each tray every two days. The CT received consistent watering of 300 ml every two days throughout the experiment. All sample types (green parts (GP), brown parts (BP), and soil) were collected across every desiccation cycle (Dc1−Dc4) and rewetting cycle (Rc1−Rc4) in all treatments. Sampling was performed on the final day of each 6-day desiccation or rewetting period.

**Figure S2 Physiological and environmental validation of control and repeated desiccation-rewetting treatment.** (A) Representative image showing phenotypic changes in *C. plumiforme* CT and DRWs treatments. The images illustrate significant leaf curling during the desiccation phase, with recovery to normal appearance after the rewetting phase. (B) Boxplots depicting tissue water content (%) in the green part (GP) of moss throughout the experimental period and across the four desiccation cycles. Significant differences determined by one-way ANOVA. NS represents *p* > 0.05. (C) Fv/Fm values in the green-part (GP) of moss, measured every two days throughout the experiment, indicating photosynthetic efficiency and recovery during the desiccation cycles (Dc), rewetting cycles (Rc), and control treatments (CT). (D) Soil volumetric water content (%) throughout the experiment, showing changes during the four desiccation and rewetting phases and in CT. (E) Water loss curves for the green-part (GP) during the four cycles of desiccation, fitted using exponential decay regression. *T* (day) is the evaporative time, and a represents the initial water status amplitude, DC (decaying constant) quantifies the rate of water loss, with higher values indicating faster dehydration and lower drought tolerance, and b are coefficients.

**Figure S3 Alpha diversity of bacterial and fungal communities across compartments and treatments.** (A) Boxplots showing the alpha diversity of both bacterial and fungal communities across different treatments among green-part (GP) and brown-part (BP) of moss and soil. The asterisks on top indicate a significant difference determined by the nonparametric Kruskal-Wallis test. ** represents *p* < 0.01, * represents *p* < 0.05, NS represents *p* > 0.05. (B) The stacked bar chart additionally illustrates the temporal dynamics of species in the GP microbiomes, with colors representing newly appeared species at each cycle (genus level). Dc: Desiccation cycle; Rc: Rewetting cycle.

**Figure S4 Temporal species turnover in the brown-part and soil microbiomes.** Bar charts displaying the temporal patterns of species composition at the genus level in the brown-part (BP) and soil samples, with colors representing newly appeared species at each time point. Dc: Desiccation cycle; Rc: Rewetting cycle.

**Figure S5 Principal coordinate analysis (PCoA) of bacterial and fungal communities across compartments and treatments, with expanded BP and soil view.** (A) PCoA based on Bray-Curtis distance, illustrating the community composition of bacteria and fungi across compartments under control treatment (CT), four desiccation cycles (Dc), and rewetting cycles (Rc). (B) PCoA was additionally performed for bacteria and fungi in each compartment, and the relative contribution of different factors on community dissimilarity was tested with PERMANOVA. “Com” represents the effect of compartment niche, and “Treat” represents the effect of different treatments, including CT, desiccation, and rewetting cycles. (C) PCoA for bacterial and fungal communities in BP and soil under four cycles of desiccation and rewetting, respectively, based on Bray-Curtis dissimilarity. Three non-parametric multivariate analyses were conducted based on Bray-Curtis dissimilarities among samples: permutational multivariate analysis of variance (Adonis), analysis of similarity (ANOSIM), and multiple response permutation procedure (MRPP).

**Figure S6 Beta diversity patterns and compositional stabilization of microbiomes across compartments.** Beta-diversity partitioning of the microbial community in different treatments indicating that the total beta diversity of bacterial and fungal communities in different treatments was mainly explained by turnover (*i.e.*, species replacement) rather than nestedness (*i.e.*, species loss). Significant differences determined by one-way ANOVA. *** represents *p* < 0.001, ** represents *p* < 0.01, * represents *p* < 0.05, NS represents *p* > 0.05.

**Figure S7 Community assembly processes in green-part, brown-part, and soil microbiomes.** (A) Violin plots showing the relative contribution of determinism and stochasticity on bacterial and fungal community assembly in the GP based on the β-Nearest Taxon Index (βNTI) values. The relative importance of five ecological processes (heterogeneous selection, homogeneous selection, dispersal limitation, homogenizing dispersal, and undominated processes) of bacterial and fungal community in GP compartment based on the βNTI and RCBray is also shown. *** represents *p* < 0.001, ** represents *p* < 0.01, * represents *p* < 0.05. (B) The relative contribution of determinism and stochasticity on bacterial and fungi assembly in BP and soil based on the βNTI values. Values of |βNTI| ≥ 2 indicate dominant deterministic processes, while |βNTI| < 2 indicate dominant stochastic processes. The percentage above and below the violin plot represented the proportion of the deterministic and stochastic processes in microbial community assembly, respectively.

**Figure S8 Temporal dynamics of bacterial-fungal interkingdom network in GP under desiccation and rewetting treatment.** The above panel represented four networks during the four desiccation cycles, while the below panel represented four networks during the four rewetting cycles. “B” and “F” represented the proportion of bacterial (red) and fungal (green) nodes, respectively; “P” and “N” represented the proportion of positive and negative edges, respectively.

**Figure S9 Temporal dynamics of bacterial-fungal interkingdom network in GP under CT treatment.** The proportions of bacterial (B) and fungal (F) nodes and positive (P) and negative (N) edges are illustrated. “B” and “F” represented the proportion of bacterial (red) and fungal (green) nodes, respectively; “P” and “N” represented the proportion of positive and negative edges, respectively.

**Figure S10 Bacterial taxonomic composition at the phylum and genus levels across compartments and treatments.** (A) Temporal dynamics of green-part (GP), brown-part (BP), and soil bacterial community composition at the phylum level. (B) Temporal dynamics of GP, BP, and soil bacterial community composition at the genus level. The genera highlighted in red represent plant beneficial bacteria (PBBs).

**Figure S11 Longitudinal clustering of bacterial genera during desiccation and rewetting phases.** Longitudinal shifts in the cumulative relative abundances of different modules that detected through hierarchical clustering in desiccation cycles (Dc) and rewetting cycles (Rc) individually. The remaining detected clusters are provided in Figure S12. Trend lines representing the mean values for each cluster throughout the four desiccation and rewetting cycles and inset donut plots display the number of genera and taxonomic composition of each module. C1−C4: cluster 1−4 based on hierarchical clustering (Ward’s algorithm) on relative abundance changes.

**Figure S12 Heatmaps and longitudinal shifts of desiccation- and rewetting-responsive bacterial modules in GP.** (A and B) Heatmap displaying the z-score scaling relative abundance between different cycles of desiccation-rewetting in GP. Each row represents ASV detected as a member of the cluster. Horizontal facets indicate each module detected through hierarchical clustering in the desiccation and rewetting cycles. (C and D) Longitudinal shifts in the cumulative relative abundances of desiccation and rewetting-responsive modules (genus level) detected through hierarchical clustering in GP. Trend lines represent the mean values for each treatment throughout the experiment, and inset donut plots display the size (number of genera) and taxonomic composition of each module. C1−C4: cluster 1−4 based on hierarchical clustering (Ward’s algorithm) on relative abundance changes.

**Figure S13 Progressive increase of plant-beneficial bacteria across desiccation cycles.** Temporal regression analysis of the abundance of plant-beneficial bacteria (PBBs) in GP across different desiccation-rewetting cycles.

**Figure S14 Correlation between plant-beneficial bacteria and community dynamics.** (A) Strong correlation between PBBs and bacterial community dynamic in GP, as indicated by Principal Coordinate Analysis (PCoA) axes. (B) The correlation between top 10 PBBs and bacterial community dynamic in GP (PCoA1).

**Figure S15 Composition of fungal communities and desiccation-responsive fungal taxa.** (A) Ternary plot displaying the differentially abundant fungal ASVs (desiccation-rewetting responsive fungi, *p* < 0.05) detected between CT and desiccation-rewetting cycles. Each circle represents one ASV, and the size of each circle represents its relative abundance. Statistical significance was determined by EdgeR’s generalized linear model (GLM) approach. (B) Heatmap showing the relative abundance of desiccation-rewetting responsive fungi over time of CT and desiccation-rewetting cycles.

**Figure S16 Functional alpha diversity of GP microbiomes across treatments.** Boxplot showing the alpha diversity of GP functions of microbial communities across different treatments. Different letters (a, b) indicate significant differences between groups (*p* < 0.05).

**Figure S17 Gene Ontology (GO) and KEGG pathway enrichment analysis for Dc4.** GO and KEGG pathway enrichment analysis for Dc4, highlighting significant functional shifts in the microbial community compared to control (CT) and Dc1.

**Figure S18 Heatmap of memory metabolites in GP.** Heatmap showing z-score scaled abundance of metabolites identified as memory metabolites in GP, highlighting significant changes in response to repeated desiccation-rewetting cycles.

**Figure S19 PCA of metabolites.** PCA of metabolites, showing a clear separation between desiccation cycle 1 (Dc1) and cycle 4 (Dc4), indicating stress-memory effects.

**Figure S20 Structural equation models (SEM) linking microbial and metabolic legacies under recurrent drought.** (A) SEM illustrating the direct and indirect effects of treatments and desiccation–rewetting cycles on microbial communities (PBBs, desiccation-rewetting responsive fungi, and microbial functional genes represented by the first PCA axis based on KOs and GOs) on the formation of memory metabolites. (B) SEM illustrating the direct and indirect effects of treatments and desiccation–rewetting cycles on moss metabolites (captured by the PCA axis) on microbial legacies, including PBBs, desiccation-rewetting responsive fungi, and functional memory genes related to element cycling. Arrow thickness represents the strength of the relationship, with solid arrows denoting significant pathways (*p* < 0.05) and dash arrows denoting non-significant pathways (*p* > 0.05). Numbers along the arrows indicated standardized path coefficients. *, **, and *** denote significance levels at *p* < 0.05, *p* < 0.01, and *p* < 0.001, respectively. Abbreviations: PBB, plant-beneficial bacteria; GOs, Gene Ontology; KOs, KEGG Orthology; GOF, goodness of fit.
